# Sequential power analysis framework in assessing social forestry outcomes

**DOI:** 10.1016/j.mex.2020.100917

**Published:** 2020-05-15

**Authors:** Muhammad Alif K. Sahide, Micah R. Fisher, Bart Verheijen, Ahmad Maryudi, Yeon-Su Kim, Grace Y. Wong

**Affiliations:** a Forest and Society Research Group, Faculty of Forestry, Universitas Hasanuddin, Makassar, Indonesia; b University of Hawai‘i at Mānoa, United States; c University of Amsterdam, Netherland; d Sebijak Institute (Research Center for Forest Policy & History Studies), Faculty of Forestry, Universitas Gadjah Mada, Indonesia; e Northern Arizona University, United States; f Stockholm Resilience Centre, Stockholm University, Stockholm SE-10691, Sweden

**Keywords:** Sequential power analysis, Power background, Power delivery, Power adjustment

## Abstract

We extend the Actor-Centred Power framework to consider dimensions beyond the life of community natural resource management partnership initiatives by examining social forestry partnership projects in Indonesia. We do this by examining how power constellations realign across the temporal phases that operationalize project partnerships. We propose a sequential power analysis framework that examines power in three parts. The framework first proposes a method for historicizing actors into their power background. Second, we present mode for examining the arrival of a partnership scheme, which we call the power delivery phase. Third, we highlight approaches for examining the way power relations are adjusted, whether reinforced or reconfigured, by introducing an approach for examining programmatic outcomes of social forestry partnership schemes. This article thus provides broadly applicable but targeted guide for the researchers collecting data and seeking to make sense of power relations on community forest partnership schemes in various contexts. This framework is particularly useful for analysing equity and justice dimensions by highlighting who benefits and who loses.

• Sequential Power Analysis (SPA) methodology is rooted in interest based and historical power framework.
• SPA is consisted of three parts: power background, power delivery, and power adjustment
• SPA framing provides a protocol for researchers to collect data

## Graphical abstract

**Figure:**
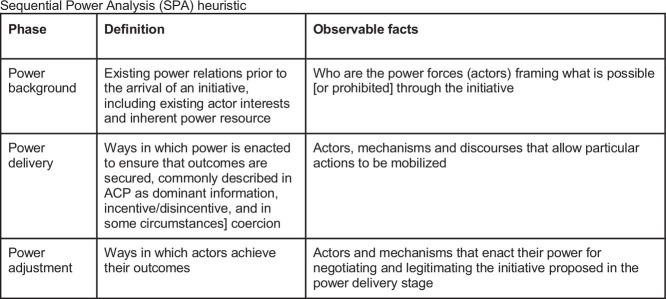

**Table:** Specifications Table

Subject Area	Environmental Science
More specific subject area	Forest policy and governance
Method name	Sequential power analysis (SPA)
Name and reference of original method	Krott, M., Bader, A., Schusser, C., Devkota, R., Maryudi, A., Giessen, L., & Aurenhammer, H. (2014). Actor–centred power: The driving force in decentralised community based forest governance. *Forest Policy and Economics, 49*, 34–42
Resource availability	Not applicable

## Method details

### Engaging historical power dimensions

Taking from behavioural theories of power [[1], [2], [3], [4], [13]], Krott et al.’s [5] Actor-centred power approach (ACP) highlights three distinct elements of power: coercion, [dis]incentives, and dominant information. By defining the observable aspects of each element, Krott et al. shows how an abstract concept of power can become empirical, and thus observable [6], [7], [8], [9], [10], [11], [12]]. However, the cases that we tried to highlight with ACP required reaching back into the past to better understand the factors that shaped these power elements. While applying ACP to Indonesia's community forest partnership schemes, we found that simply teasing apart the existing power dynamics is not enough, but we also need to firmly foreground the historical context where current elements of power relations have emerged. In this paper, we present a framework that we call a Sequential Power Analysis (SPA), which is a three-part analysis for understanding power.

SPA begins with an overall examination of the pre-existing dimensions, which we call the *power background*. At this stage, the focus is on understanding the historical contexts of how various actors have competed to frame what is possible in governing a landscape. The second stage is around the *delivery of power* on governing a particular initiative. The focus is on understanding how the actors identified in the power background extend their involvement across the different aspects of an initiative to negotiate power relations. In the third and finally stage, we examine the outcomes achieved, which actors are involved and which mechanisms (regulations, budgets, projects, etc.) are employed to *adjustment power.* Table 1 provides a more mechanistic representation of our SPA framework.

**Table 1:** Sequential power analysis (SPA) heuristic.

Phase	Definition	Observable facts
Power background	Existing power relations prior to the arrival of an initiative, including existing actor interests and inherent power resource	Identifying the powerful actors representing their interests in the framing of what is possible [or prohibited] in all initiatives
Power delivery	Ways in which power is enacted to ensure that outcomes are secured, commonly described in ACP as dominant information, incentive/disincentive, and in some circumstances] coercion	Actors, mechanisms and discourses that allow particular actions to be mobilized
Power adjustment	Ways in which actors achieve their outcomes	Actors and mechanisms that enact their power for negotiating and legitimating the initiative proposed in the power delivery stage

### Protocols: what should researchers be aware of when collecting data for SPA?

Table 1 describes the stages/elements of our power analysis, alongside the overall definition and the observable dimensions guiding our framework. We provide some simple protocols to guide researchers on collecting data for SPA, which is presented in Table 2

**Table 2:** Protocol on collecting data for SPA.

	Power background	Power delivery	Power adjustment
Observing the source of power	(i) historical and socio-cultural factors, (ii) existing powerful actors and institutions, (iii) regulatory power, and (iv) market-driven power	(i) actors who maintain powerful influences, (ii) delivery actions, such as programs and initiatives (iii) coalitions and oppositions (iv) agreements	(i) Negotiation process and mechanisms, (ii) actions for legitimizing the initiative, (iii) practical outcomes from the negotiation (e.g. benefit- and cost- sharing)
Influential pattern	Power elements that arise from previous power relations amongst actors and their roles, dominant and opposition	The first group of actors (who deliver on the initiative) and their success in taking actions such as introducing programs, which open up the opportunities for collaboration.	Actors who maintain the main interests by deploying all means at hand to influence others; actors who resist or contest such initiatives
How to collect data (A general guide on collecting data)	− Observations: It is best to start with observations of historical power relations through understanding how political instruments were designed and used in the past by asking why, when, and whose interests were served [and whose were not]− Document Analysis: Policy documents, previous research, and programmatic reports can provide historical contexts of power.− Interviews: To validate observations and document analysis results.	− Observations: Seek access to be part of participants on delivery action processes, which provides a very good opportunity to directly observe interests of actors and power they use to influence others and to quell dissent.− Interviews: Researcher should be aware of the contexts of the programmatic/regulatory delivery action.− Interview relevant actors involved in programmatic/regulatory delivery, as well as the market driven channels, and other non-state facilitated activities that are usually developed on behalf of powerless actors.− Policy and project document analysis: These documents highlight the overall priorities that continue to justify the project and describe its progress, which are valuable markers for analysis.	− Observation: Actors who maintain the interests in the main political agenda; those who hide important information or spreading unverified information; those who offer incentives-disincentives; those who deploy coercive means; those who resist or provide alternative narratives− Interviews: Tracking back the previous agreements resulted from the delivery action, ask relevant actors what promises have been delivered and what have not, why and how?− Policy and project document analysis: similar to the examination of dimensions of power delivery, examining project documentation helps to highlight the key turning points that justify or nullify new actions

Supplementary material *and/or Additional information: -*
